# Directed cardiomyogenesis of autologous human induced pluripotent stem cells recruited to infarcted myocardium with bioengineered antibodies

**DOI:** 10.1186/2052-8426-2-13

**Published:** 2014-05-01

**Authors:** Marek Malecki, Emily Putzer, Chelsea Sabo, Afsoon Foorohar, Carol Quach, Chris Stampe, Michael Beauchaine, Xenia Tombokan, Raf Malecki, Mark Anderson

**Affiliations:** Phoenix Biomolecular Engineering Foundation, San Francisco, CA USA; National Magnetic Resonance Facility, National Institutes of Health, Madison, WI USA; University of Wisconsin, Madison, Madison, WI USA; Latin American Youth Center, Washington, DC USA; University of Sheffield, Sheffield, EU UK; Western University, Lebanon, OR USA; Western University, Pomona, CA USA; University of Minnesota, Minneapolis, MN USA; AXS Bruker, Madison, WI USA; BioSpin Bruker, Woodlands, TX USA; San Francisco State University, San Francisco, CA USA

**Keywords:** Myocardial infarction, Cardiac regeneration, Stem cell therapy, Recruitment and retention of stem cells, Autologous human induced pluripotent stem cell, Heterospecific, Tetravalent antibodies, Stage specific embryonic antigen, Tumor related antigen, Induced pluripotent stem cell tumorigenicity

## Abstract

**Objective:**

Myocardial infarctions constitute a major factor contributing to non-natural mortality world-wide. Clinical trials ofmyocardial regenerative therapy, currently pursued by cardiac surgeons, involve administration of stem cells into the hearts of patients suffering from myocardial infarctions. Unfortunately, surgical acquisition of these cells from bone marrow or heart is traumatic, retention of these cells to sites of therapeutic interventions is low, and directed differentiation of these cells *in situ* into cardiomyocytes is difficult. The specific aims of this work were: (1) to generate autologous, human, pluripotent, induced stem cells (ahiPSCs) from the peripheral blood of the patients suffering myocardial infarctions; (2) to bioengineer heterospecific tetravalent antibodies (htAbs) and use them for recruitment of the ahiPSCs to infarcted myocardium; (3) to initiate *in situ* directed cardiomyogenesis of the ahiPSCs retained to infarcted myocardium.

**Methods:**

Peripheral blood was drawn from six patients scheduled for heart transplants. Mononuclear cells were isolated and reprogrammed, with plasmids carrying six genes (*NANOG, POU5F1, SOX2, KLF4, LIN28A, MYC*), to yield the ahiPSCs. Cardiac tissues were excised from the injured hearts of the patients, who received transplants during orthotopic surgery. These tissues were used to prepare *in vitro* model of stem cell therapy of infarcted myocardium. The htAbs were bioengineered, which simultaneously targeted receptors displayed on pluripotent stem cells (SSEA-4, SSEA-3, TRA-1-60, TRA-1-81) and proteins of myocardial sarcomeres (myosin, α-actinin, actin, titin). They were used to bridge the ahiPSCs to the infarcted myocardium. The retained ahiPSCs were directed with bone morphogenetic proteins and nicotinamides to differentiate towards myocardial lineage.

**Results:**

The patients’ mononuclear cells were efficiently reprogrammed into the ahiPSCs. These ahiPSCs were administered to infarcted myocardium in *in vitro* models. They were recruited to and retained at the treated myocardium with higher efficacy and specificity, if were preceded the htAbs, than with isotype antibodies or plain buffers. The retained cells differentiated into cardiomyocytes.

**Conclusions:**

The proof of concept has been attained*,* for reprogramming the patients’ blood mononuclear cells (PBMCs) into the ahiPSCs, recruiting these cells to infarcted myocardium, and initiating their cardiomyogenesis. This novel strategy is ready to support the ongoing clinical trials aimed at regeneration of infarcted myocardium.

## Background

Myocardial infarctions (MI) constituted a major factor contributing to non-natural mortality world-wide in 2012 [1, 2].

Causes of myocardial infarctions are complex, but all result in cessation of blood supply to and necrosis of myocardium. Subsequently, some of the sarcomeric proteins are quickly and freely released from the infarcted myocardium into blood circulation. They become diagnostic biomarkers of myocardial damage detected with antibodies in blood and urine, e.g., antibodies against troponin or myosin light chains used for enzyme-linked immunosorbent assay (ELISA). The remaining cardiac proteins are retained within sarcomeres, but are exposed to constituents of blood through the damaged endothelia and sarcolemmas. They become landmarks of location and extent of the cardiac damage determined by antibody-guided contrast agents for molecular imaging, e.g., antibodies to myosin heavy chains modified with radionuclide for positron emission tomography (PET) or modified with superparamagnetic clusters for magnetic resonance imaging (MRI). Immediate surgical revascularization is critical to prevent progression of myocardial injury, which otherwise may result in heart failure and the patient’s death [3–5].

Clinical trials of myocardial regenerative therapy, currently pursued by cardiac surgeons, involve administration of cells, which have ability to differentiate or trans-differentiate into cardiac muscle or endothelia, into the hearts of patients suffering myocardial infarctions [5–8]. The rationale for this strategy is to replace necrotic cardiac muscle with new, functional cells. Main routes of delivery involve: myocardial injections, blood infusions, or pericardial patches [5–9]. However, recruitment and retention of these cells to the sites of therapeutic interventions is so low, that only 1-3% of the infused and 6-12% of the injected cells are detected at the sites of therapeutic deliveries 2 weeks later [5–9]. This translates into poor and inconsistent therapeutic effects. Therefore, resolving this problem has been recognized as the most critical priority to achieve progress in stem cell-based cardiac therapy [5–11]. Attempts to compensate for the losses in administered cells by increasing their numbers are limited by the injections’ volumes [9]. Therefore, the novel biotechnology has been developed to improve recruitment and retention of therapeutic stem cells with the aid of bioengineered, heterospecific, polyvalent antibodies (htAbs), which efficiently anchor more than 90% of administered cells to the regenerated myocardium [10, 11]. These antibodies are uniquely specific for the human stem cell stage specific antigens resulting in acquisition of the data that are clinically relevant and ready for streamlining into clinical trials. This feature is essential, since the biomarkers of human stem cells are different, expressed at different stages of differentiation, or completely absent, than those of non- humans. The htAbs demonstrate exquisite specificity and affinity, which after their fluorescent, elemental, or magnetic modification; ensure selection of batches of pluripotent stem cells with high purity and viability, as well as tracking these cells in vivo [12–18].

Human, bone marrow cells are most commonly used in the ongoing clinical trials of cells-based cardiac regeneration [5–10]. Cardiac stem cells have also been recently introduced into the clinical trials [5–9]. Human induced pluripotent stem cells, which are reprogrammed from bone marrow, heart, skin, or peripheral blood are vigorously studied [11, 19–27]. Alternatively, cells differentiating towards cardiac cells are generated by directed lineage reprogramming [10, 28–38].

In general, the main problem with the therapeutic efficacy of cells-based therapy is still the recruitment and retention of the administered stem cells to the sites of the intended therapeutic interventions [5–9, 39, 40]. Moreover, the challenge for using stem cells in regenerative medicine is to assure their differentiation or trans-differentiation towards the desired lineage and functional integration with the host tissue. Finally, the most serious problem with streamlining the pluripotent stem cells into clinics is the risk of their tumorigenic transformation [41–50].

In this realm, the specific aims of this work were: (1) to generate autologous, human, pluripotent, induced stem cells (ahiPSCs) from the peripheral blood of the patients suffering myocardial infarctions; (2) to bioengineer heterospecific antibodies (htAbs) and use them for recruitment of the ahiPSCs to the infarcted myocardium; (3) to initiate *in situ* directed cardiomyogenesis of the ahiPSCs retained to infarcted myocardium.

## Methods

### Concept for recruitment and retention of pluripotent induced stem cells to infarcted myocardium with bioengineered antibodies

**Figure 1:**
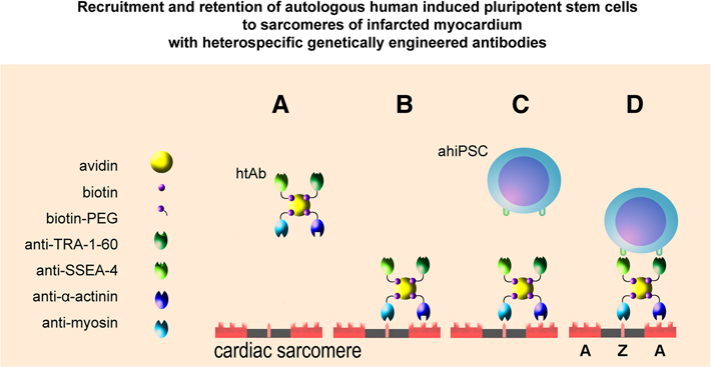
**Concept for recruitment of human pluripotent stem cells to sarcomeres of infarcted myocardium.** Sarcomeric proteins include: myosin (in A-band), α-actinin (in Z-line), actin (I-band), titin (with various domains stretched between Z-lines). Autologous human induced pluripotent stem cells (ahiPSCs) display on their surfaces biomarkers: SSEA-4, TRA-1-60, SSEA-3, TRA-1-81. Heterospecific tetravalent antibodies (htAbs) target the sarcomeric proteins and simultaneously the pluripotent stem cells’ biomarkers. Four phases of recruitment are shown: (**A** – injection of htAbs, **B** – anchoring to sarcomeres; **C** – injection of ahiPSCs; **D** – anchoring of ahiPSCs). For clarity of the presentation, only bonds between myosin, α-actinin and TRA-1-60, SSEA-4 are shown.

Principles of a novel strategy, for anchoring autologous, human, pluripotent, induced stem cells (autologous hiPSCs or ahiPSCs) to sarcomeres of infarcted myocardium, with the aid of the bioengineered, heterospecific tetravalent antibodies (htAbs), are illustrated (Figure 1). These principles are applicable to an *in vitro* model of regenerative therapy developed in this work, as well as to potential streamlining into clinical trials *in vivo*. Genetically engineered, monovalent synthetic nano-antibodies are against pluripotent stem cells’ biomarkers: SSEA-4, SSEA-3, TRA-1-60, TRA-1-81, and simultaneously against sarcomeric molecules: myosin, α- actinin, actin, and titin. Each nano-antibody is modified to carry a single biotin group at the carboxyl terminus. One at a time, these antibodies are docked into avidin. This way they form sequentially batches of mono-, bi-, tri-, or tetra-valent antibodies.

Our novel “recruit-and-retain” therapeutic strategy consists of four phases. First, htAbs are spiked into the solution flowing over sarcomeres (A). The htAbs are recruited onto the sarcomeres through their anti-myosin, anti-α-actinin, anti-actin, and anti-titin domains. (B). After clearing out unbound htAbs, the solution is spiked with SSEA-4, SSEA-3, TRA-1-81, and TRA-1-60 displaying autologous hiPSCs (ahiPSCs) (C). The ahiPSCs dock into the anti-SSEA-4, anti-SSEA-3, anti-TRA-1-81, and anti-TRA-1-60 domains of the htAbs, which remain anchored onto the sarcomeres (D). Thereafter, these anchored autologous, human, pluripotent, induced stem cells are subjected to directed differentiation towards the heart regenerating cardiomyocytes.

### Patients

All samples were obtained in accordance with the Declaration of Helsinki with the Institutional Review Boards’ Approval and with the Patients’ Informed Consent. The cohort consisted of 3 men and 3 women, who after suffering from multiple infarctions, were recommended to receive orthotopic heart transplants and agreed to using infarcted myocardium for research.

### Peripheral blood mononuclear cells

Small volumes of blood (1-10 ml) were drawn from these patients according to standard clinical laboratory practices into heparin tubes (green tops). Blood was diluted 2-4x with physiological buffered saline (PBS) without calcium and magnesium in sterile tubes and layered over Ficoll. That followed by centrifugation in swinging bucket rotor at 400xg, 22°C for 30 min. Plasma layers were transferred to separate tubes. Buffy coats containing PBMCs were gently aspirated, suspended in fresh PBS and spun at 350xg, 22°C for 10 min. After discarding supernatants, the cells were suspended in PBS again and spun down at 200xg, 22°C for 10 min. [14, 15, 18–20]. Approximately 5 million of mononuclear cells were isolated from 10 ml of blood. These cells were immediately processed as described below. Alternatively, they were suspended in PBS supplemented with 5% starch, 5% DMSO, 30% human serum for 15 min. on ice and cryoimmobilized in the programmable freezer (the freezer was designed and built based upon the NSF funds granted to Dr M. Malecki, Principal Investigator) at 1°C/min down to -30°C at 1°C/min, rapid cooling between down to -70°C at 30°C/min, and the final phase down to -196°C at 3°C/min.

### Human autologous pluripotent induced stem cells

Mononuclear cells from peripheral blood were reprogrammed into human, autologous, pluripotent, induced stem cells (autologous hiPSCs or ahiPSCs) by transduction with plasmids in biotags carrying six genes *OCT4, SOX2, NANOG, LIN28, KLF4, MYC* in media supplemented with 1 mM valproic acid (VPA), 1 mM antibody to transforming growth factor-β receptor 1 (anti-TGFR1). The plasmid vectors carried chelating domains, which permanently tagged the stem cells. Sustained cultures of the autologous hiPSCs and human embryonic stem cells (hESCs) were grown in Dulbecco’s Modified Eagle’s Medium (DMEM) supplemented with knockout serum replacement (KOSR), mercaptoethanol, glutamine, nonessential amino acids, fibroblast growth factor 2 (FGF2). They were subjected to three rounds of enrichment by magnetic or fluorescent activated cell sorting to attain > 99% purity. That followed by 50–100 fold clonal expansion and long term cultures in CelliGen BLU Single-Use, Stirred-Tanks Bioreactors (New Brunswick, NJ, USA) with the batch media feeding, impeller set at 100 rpm, and all USP Class VI and animal component free materials, thus GMP compliant, as described [14–16, 26–28]. Pluripotency of these cells was determined by detecting cell surface display of biomarkers and ability to form embryoid bodies (EBs). Cell surface displayed biomarkers were quantified and isolated by fluorescence and magnetic activated sorting after labeling with fluorescent and superparamagnetic antibodies’ (respectively) against: SSEA-4, SSEA-3, TRA-1-60, TRA-1-81, which were thoroughly characterized [17, 18].

Ability to form the EBs was determined by transferring onto poly(2-hydroxyethyl-methacrylate)-coated dishes in media 20% knockout serum replacement (Invitrogen, Carlsbad, CA, USA), L-glutamine, nonessential amino acids, mercaptoethanol, penicillin, streptomycin in DMEM/F12 exchanged 3x for a week. After a week, the individual EBs were transferred into matrigel-coated dishes in the same media for another week. Differentiation was determined by measuring transcripts by qPCR and products by immunocytochemistry for genes unique for the three main germ layers. Quantitative analysis *in situ* of differentiation kinetics was facilitated by labeling with antibodies against myosin heavy chains, neurofilamentous proteins, cytokeratins, adrenergic β1 receptors, acetylcholine receptors, and platelet endothelial cell adhesion molecules, which were modified with: (1) superparamagnetic clusters, so that they were affecting relaxivities of the labeled samples in NMRS; (2) elemental tags, so that they were changing the scintillation counts radiating from the labeled samples in EDXS or XRFS [15]. Both approaches save sample preparation times, are much safer, and easier to implement for academic laboratories.

### Cardiac tissues

Cardiac tissues were sampled from the infarcted hearts, while the transplants’ recipients were undergoing orthotopic procedures. The tissues were transferred into the University of Wisconsin solution immediately after the release from thorax. They were processed within 4 h or cryoimmobilized for storage in liquid nitrogen at -196°C indefinitely (the freezer was designed and built based upon the NSF funds granted to Dr M. Malecki, PI).

Immediate processing involved five routes. (1) Native myofibrils were prepared to retain antigenicity of sarcomeric proteins. Strips of cardiac muscle tissue were immersed in myofibril buffered solution: 75 mM KCI, 10 mM Tris pH6.8, 2 mM EGTA, 2 mM MgCl2, 0.1 mM PMSF, 0.1% TritonX-100 and homogenized in Polytron (Brinkman Instruments Co., Westbury, NY, USA) and Teflon glass homogenizer. That followed by three cycles of spinning and suspending. They were immediately used to assemble models of infarcted myocardium inside environmental chambers or infused with the fresh myofibril buffered solution containing 50% glycerol and frozen. (2) Cryosections were cut from the cardiac tissues, which were rapidly cryoimmobilized in the HPM 010 (Balzers, Lichtenstein, EU) for retaining life-like antigenicity. The frozen muscles were either sectioned directly in frozen hydrated state or cryo-substituted, infused with 2.3 M sucrose, refrozen, then sectioned (Leica, Vienna, A, EU). (3) The frozen tissues were crushed, homogenized, and lyophilized to be used, after thawing and dialysis, for immunoblotting or supplementing cell cultures. (4) The cardiac tissues were homogenized and dissociated in denaturing sample solution: 1% sodium-dodecylsulafate, 10% glycerol, 10 mM Tris-Cl, pH 6.8, 1 mM ethylene-diamine tetraacetic acid, with or without reducing agents 2 mg/ml dithiothreitol (DTT) or 2- mercaptoethanol, colored with ~0.05 mg/ml bromophenol blue. (5) Cell cultures were initiated from the cardiac tissues and grown in incubators maintaining 37°C, 10% CO2, and saturated humidity.

Cryoimmobilization was accomplished in the programmable freezer (the freezer was designed and built based upon the NSF funds granted to Dr M. Malecki, PI) at 1°C/min down to -30°C at 1°C/min down, rapid cooling down to -70°C at 30°C/min, and the final phase down to -196°C at 3°C/min. It was conducted after equilibrating the tissue with 5% starch, 5%DMSO, 30% human serum in PBS for 15 min.

All samples were examined by multiphoton fluorescence spectroscopy (MPFS), immunoblotting (IB), flow cytometry (FCM), quantitative reverse transcription and polymerase chain reaction (qRTPCR), nuclear magnetic resonance spectroscopy (NMRS), energy dispersive x-ray spectroscopy (EDXS), and x-ray fluorescence spectroscopy (XRFS) as described below.

### Bioengineering of heterospecific tetravalent antibodies (htAbs)

Heterospecific tetravalent antibodies were bioengineered using coding sequences from two groups of the nano-antibodies developed earlier, which were targeting: pluripotency biomarkers and sarcomeric proteins as described [15–18, 39, 40].

The B cells were collected from the patients suffering myocardial infarctions, as well as from the patients suffering ovarian and testicular embryonal carcinomas. The mRNA was isolated (Trizol, MRC, Cincinnati, OH, USA) and reverse transcribed to cDNA (Cell-to-DNA, Qiagen, San Diego, CA). These new sequences were assembled into the libraries of anti-cancer-antibodies (ACA) coding sequences and anti-heart antibodies (AHA). The cds, after insertion into the plasmids containing chelates’ harboring coding sequences under the CMV promoters and terminated with polyA, were propagated and expressed in human myelomas.

Complementarity determining regions (CDR) and framework regions (FWR) were re-engineered by sequence shuffling, while expressed on human myelomas. The native SSEA-4, SSEA-3, TRA-1-60, TRA-1-81, CD-45, CD-34, CD-19, CD-20, myosin, actin, titin, and α-actinin were purified by immunoprecipitation with monoclonal antibodies, which followed by their modification with biotin, digoxigenin, tetramethylrhodamine, or fluorescein. They were anchored onto anti-biotin, anti-dig, anti-TMR, or anti-FITC saturated pans and served as baits for selection of the antibodies clones from the aforementioned libraries. The chelates were saturated with Ni, Co, Gd, Fe, Tb, and Eu as described. The specificity and sensitivity were determined based upon elemental compositions with EELS (Zeiss, Oberkochen, D, EU), EDXS (Noran, Middleton, WI, USA), or XRFS (Bruker AXS, Fitchburg, WI, USA). The fluorescent properties were measured with the RF-5301PC spectrofluorometer (Shimadzu, Tokyo, Japan). The specificity and sensitivity of the antibodies were tested with the EELS and EDXS. The magnetic relaxivities were measured on the DMX 400 WB or AVANCE II NMR spectrometers (Bruker Optics, Dallas, TX, USA). The monoclonal antibodies for these antigens served as the positive controls, and antibodies towards 6His, DOTA, TETA, and DTPA served as the negative controls.

For preparing tetravalent antibodies, the first batch of monovalent antibodies was sprayed from the air-brush with a single pulse over the pan filled with the 0.01-0.001 mg/mL recombinant avidin (rA) in PIPES buffer in a saturated humidity chamber maintained at room temperature. Upon completion of binding, the fractions of resulting solution were separated by the size exclusion chromatography on high pressure liquid chromatography columns (HPLC) (Pharmacia, S, EU) with 0.5 mL/min flow at 30 bar pressure. The fractions were collected on the fraction collector equipped with the 280 nm sensor (Pharmacia, S, EU). The fractions detected at the ~95kD peak and containing rA linked with the single monovalent antibody were pooled together and filled a new pan. That was uniformly sprayed over with the new antibodies. The first peak of rA saturated with four monovalent antibodies was used for specific blocking on competitive assays. The procedure was repeated for all antibodies, one at a time, in a random order. In the final separation tetravalent antibodies were collected at the peak of ~170 kDa. The system was calibrated using classic IgG, BSA, avidin, Fab, and myoglobin as the standards. Specificity and sensitivity of the bioengineered tetravalent antibodies were tested by immunoblotting (IB), labeling and multiphoton fluorescence spectroscopy (MPFS), EDXS, and EELS.

### Flow cytometry (FCM), Fluorescently activated cell sorting (FACS), Multiphoton Fluorescence Spectroscopy (MPFS)

For flow cytometry (FCM), fluorescent activated cell sorting (FACS) and magnetic activated cell sorting (MACS) the cells were thoroughly prepared as single cell suspensions by short treatment with the PIPES buffered DNase, RNase, trypsin, collagenase, or dispase II (0.5 units/mg). These preparations included cell clusters and embryoid bodies. Moreover, apoptotic cells were removed with the anti-PS and dead cells with the anti-DNA antibodies. The enriched populations of the cells were labeled with the anti-TRA-1-60, anti-TRA-1-81, anti-SSEA-3, and anti-SSEA-4 fluorescent antibodies, which were thoroughly characterized previously [17, 18].

They were quantified with the Calibur, Vantage SE, or Aria (Becton-Dickinson, Franklin Lakes, NJ, USA) or the FC500 (Beckman- Coulter, Brea, CA, USA). The fluorescently labeled cells were imaged with the Axiovert (Zeiss, Oberkochen, D, EU) equipped with the Enterprise argon ion (457 nm, 488 nm, 529 nm lines) and ultraviolet (UV) (364 nm line) lasers; Odyssey XL digital video-rate confocal laser scanning imaging system operated up to 240 frames*/*s under control of Intervision software (Noran, Madison, WI, USA), and the Diaphot (Nikon, Tokyo, Japan) equipped with the Microlase diode-pumped Nd:YLF solid state laser (1048 nm line), the pulse compressor with the pulses’ rate 300 fs at 120 MHz and the MRC600 scanning system under control of Comos software (the multi-photon fluorescence station built based upon the NIH funds for Dr J. White, PI). Deconvolution of images was done on the Indy workstation (Silicon Graphics, Fremont, CA, USA).

### Nuclear magnetic resonance spectroscopy (NMRS) Magnetic activated cell sorting (MACS)

The cells were labeled for positive selection with the superparamagnetic antibodies targeting TRA-1-60, TRA-1-81, SSEA-3, and SSEA-4, and for the negative selection targeting CD45, CD34, dsDNA, and PS, while suspended in the physiological buffer supplemented with serum and glucose. The small aliquots were dispensed into the magnetism-free NMR tubes (Shigemi, Tokyo, Japan). The relaxation times T1 were measured in resonance to the applied FLAIR pulse sequences on the NMR spectrometers: DMX 400 WB or AVANCE II NMR (Bruker, Billerica, MA) or the Signa clinical scanners (GE, Milwaukee, WI, USA). The superparamagnetic antibodies were also used to isolate the labeled cells from the solution using the magnetic sorter to reach above 99.5% of purity (the sorter designed and built based upon the NSF funds for Dr M. Malecki, PI).

### Electron energy loss spectroscopy (EELS), Energy dispersive x-ray spectroscopy (EDXS), X-ray reflection fluorescence spectroscopy (XRFS)

The samples, which were cryo-immobilized, presented the life-like supramolecular organization. Molecular imaging was pursued as described. The field emission, scanning transmission, electron microscope FESTEM HB501 (Vacuum Generators, Kirkland, WA, USA) was equipped with the energy dispersive x-ray spectrometer (EDXS) (Noran, Middleton, WI, USA) and post- column electron energy loss spectrometer (EELS) (Gatan, Pleasanton, CA). The cryo-energy filtering transmission electron microscope 912 Omega was equipped with the in-column, electron energy loss spectrometer (EELS) (Zeiss, Oberkochen, D, EU). The cryo-energy filtering transmission electron microscopes 410 and 430 Phillips were equipped with the post-column, electron energy loss spectrometers (EELS) (Noran, Middleton, WI, USA). The field emission, scanning electron microscope SEM1530 (Zeiss, Oberkochen, D, EU) was equipped with the energy dispersive x-ray spectrometer (EDXS) (Noran, Middleton, WI, USA). The field emission, scanning electron microscope 3400 was equipped with the energy dispersive x-ray spectrometer (EDXS) (Hitachi, Tokyo, Japan). The images and spectra were acquired using the ccd camera operating under the image acquisition and processing software (SIS, Herzogenrath, D, EU or Emispec Systems, Tempe, AZ, USA).

In the XRFS study, the ICP standard of 1000 mg/l of mono-element Gallium (CPI International, Denver, CO, USA) was added to 500 microL of each sample to the final concentration of 10 mg/l. The data were generated from the S2 Picofox XRF spectrometer equipped with a molybdenum (Mo) X-ray target and the Peltier cooled Xflash Silicon Drift Detector (Bruker AXS, Fitchburg, WI, USA). Scan times ranged up to 1000 seconds. The automatic sample changer, which can hold up to 25 samples, was also used along with the SPECTRA 7 software for the instrument control, data collection, and analysis (Bruker AXS, Fitchburg, WI, USA).

### Immunoblotting (IB)

The cells and tissues were either frozen in liquid nitrogen, crushed, and thawed or disintegrated with ultrasonicator (Branson Ultrasonic, Danbury, CT, USA) within the sample buffers for native protein analysis. They were stored in liquid nitrogen or electrophoresed in the native buffer (Invitrogen, Carlsbad, CA, USA). They were vacuum or electro-transferred onto the PVDF membranes (Amersham, Buckinghamshire, UK, EU). The membranes carrying the transferred proteins were soaked within human serum and labeled with the antibodies. The samples of purified cardiac muscle myosin, actin, α-actinin, titin served as the controls.

The commercially available monoclonal antibodies against myosin, actin, α-actinin, titin, SSEA-3, SSEA-4, TRA-1-60, TRA-1-81, CD34, and CD45 served as the controls. The images of the blots were acquired and quantified with Fluoroimager (Molecular Dynamics, Sunnyvale, CA, USA) or Storm 840 (Amersham, Buckinghamshire, UK, EU). The levels of the gene expression products were also calculated, as the ratio between the protein concentration in the examined patient’s cells and the controls.

### Quantitative reverse transcription and polymerase chain reaction (qRTPCR)

Total RNA was isolated with TRIzol (MRC, Cincinnati, OH, USA). In addition to the patients’ cardiac tissues, the fibroblasts, peripheral blood cells, and bone marrow cells were processed. The cultured fibroblasts (IMR90), human embryonic stem cells (H1, H9), and blood from the healthy volunteers served as the controls. For all, RNA served as the template to generate cDNA through reverse transcription using random hexamers and reverse transcriptase (ABI, Foster City, CA, USA). The primers’ sequences and cycling settings were published [14–18]. The transcripts for GAPDH and actin served as the internal controls (ABI, Foster City, CA, USA). They were synthesized on the 380A DNA Synthesizer (ABI, Foster City, CA, USA). The PCR reactions were carried using the mix of the cDNA, the synthesized primers, dNTPs, and Taq DNA polymerase (Hoffmann–La Roche, Basel, H) on the Robocycler (Stratagene, San Diego, CA, USA), Mastercycler (Eppendorf, Hamburg, D, EU), or 7500, 7900 systems (ABI, Foster City, CA, USA). The images of the gels were acquired and quantified with Fluoroimager (Molecular Dynamics, Sunnyvale, CA, USA) or Storm 840 (Amersham, Buckinghamshire, UK, EU). The levels of the transcripts were all normalized against GAPDH or actin. Thereafter, they were calculated as the ratios between the transcripts’ concentration in the examined patient’s cells versus the cells from the healthy control tissues and cultures.

### Targeting and retention of pluripotent stem cells to cardiac sarcomeres

Simulation of the therapeutic procedure for delivering of the stem cells to the infarcted sarcomeres was based upon the models of the myocardial infarctions prepared in the environmental chambers. The bottoms of these chambers were covered with firmly attached native myofibrils or sectioned cardiac infarcted tissues. Alternatively, the human artery endothelial cells were grown as monolayers at the bottom of the chambers to imitate the coronary artery walls. In both cases the sarcomeres were exposed to the over-flowing solution as they would be in the myocardial infarctions. The buffered culture medium was flown over these sarcomeres, while propelled by the peristaltic pump (Flowrox, Linthicum, MD, USA). The chambers were tightly sealed and connected with the environmental incubator through flexible Tygon hoses. They assured maintaining the myofibrils and cells at 37°C, pH 7.3, 120/80 mmHg, and 330 mOsm. Under continuous flow, the buffer was spiked with the htAbs against myosin, actinin, titin, and actin. After, 30 minutes, the circulating htAb solution was replaced with the new, htAb-free solution. Thereafter, the induced pluripotent stem cells were spiked in. At various time intervals, the flow was stopped and the number of cells evaluated based upon quantifying changing ratios in relaxivities or x-ray scintillations. These quantifications were possible due to the superparamagnetic or elemental tags carried by the cells [14–18].

### Directed cardiomyogenesis of pluripotent stem cells

Pluripotent stem cells were anchored to sarcomeres in the environmental chambers described above. The circulating solution consisted of Dulbecco’s Modified Eagle’s Medium supplemented with 20% human serum, 10% lyophilized cardiac tissue, while maintained at 37°C and controlled O2/CO2/N2 ratios. For triggering cardiomyogenesis that solution was supplemented with 10 ng/ml bone morphogenetic protein (BMP), 10 mM nicotinamide (NAM), and antibodies 10 ng/ml anti**‒**fibroblast growth factor receptor (anti-FGFR), 10 ng/ml anti- vascular endothelial growth factor receptor (anti-VEGFR), 10 ng/ml anti-platelet derived growth factor receptor (anti-PDGFR), which were preceded by 24 h pre-treatment with media supplemented with 100 ng/ml hrActivin A (Act) and 100 ng/ml hr-Wingless-related integration site gene product (Wnt3) (R&DS, Minneapolis, MN, USA). Differentiation versus pluripotency was determined on the 1st, 5th, and 12th day, by qRTPCR of transcripts for genes: *GATA4, MEF-2c*, *OCT4, NANOG*[22, 23].

### Proliferation and differentiation of cells in embryoid bodies

Single cardiomyocytes were isolated from early (day 5–12) and late (day 59–62) embryoid bodies. From contracting embryoid bodies the single cardiomyocytes were cultured in the media supplemented with 10 μM BrdU (Invitrogen, Carlsbad, CA, USA). Cells were fixed in 4% formaldehyde, permeabilized in 0.1% Triton X-100 and denatured in 2 mol/L HCL in PBS for 30 minutes at room temperature. The cells were labeled with anti-BrdU, anti-dsDNA, and anti-cardiac myosin synthetic antibodies described above [39] which were modified with metal binding domains chelating Tb, Gd, or Eu. The ratios of BrdU positive cells were calculated in relation to the number of nuclei of cardiac myosin positive cells.

### Statistical analysis

The statistical analysis and presentations were performed using GraphPad Prism software (GraphPad Software, Inc, San Diego, CA, USA). All the data were acquired from at least three independent runs of each patient’s samples. For comparisons between two groups of data, the unpaired *t*-test was applied and *P* calculated. The statistics were calculated and presented as mean ± standard deviation of the mean. The results were considered as statistically significant for *P* < .05.

## Results

### Preservation of antigenicity and architecture in infarcted myocardial sarcomeres

**Figure 2:**
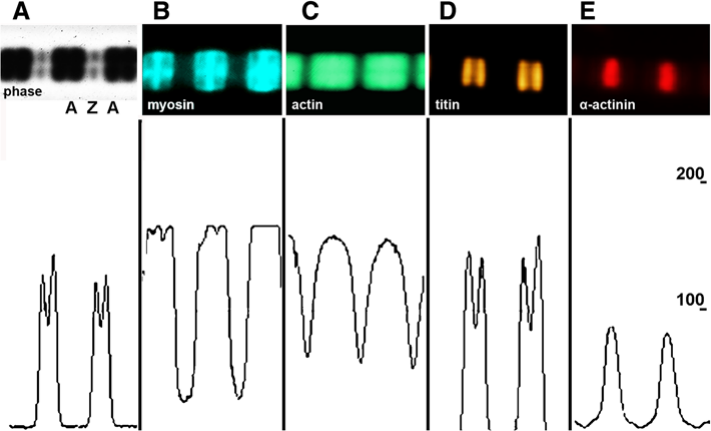
**Molecular architecture of sarcomeres in infarcted myocardium. (A)** Preservation of the myofibril structure was imaged by phase contrast light microscopy (Z = Z-line; A = A-band). Underneath the image, there is the corresponding, quantified analysis of the spatial distribution of the optical density (Y axis - relative units). It is a way to estimate sarcomers’ structural integrity. **(B-E)** Distribution of proteins was determined with the htAbs carrying chelated atoms of exogenous elements and emitting specific spectra. Colors were assigned artificially. Underneath the images, there are quantifications of spatial distribution of fluorescence intensity. Correlations between **A** and **B**-**E** determine, if the molecules retain their positioning within the sarcomeres’ architecture. HFW: 4.8 μm. Pixel brightness compared between the specific labels and the backgrounds during acquisition was accepted at the statistical significance *P* < .0001.

Preservation of structural integrity of sarcomeric architecture in infarcted myocardium was determined with Zernicke’s phase-contrast imaging of myofibrils (Figure 2). This followed by analysis of the pixel density distribution. The main framework of sarcomeres was preserved with solid A-bands and clearly marked Z-lines. These structures retained sharp edges indicative of complete absence of proteolytic degradation.

Retention of antigenicity of sarcomeric proteins in infarcted myocardium was validated by labeling with tetravalent antibodies. Each antibody was specific for a particular sarcomeric protein and was tagged with a different fluorochrome. This facilitated multiple labeling. The images of the same myofibril, as that shown by Zernicke’s phase contrast, were acquired by multiphoton fluorescence spectroscopy. That facilitated correlations between architecture and antigenicity. Multiple labels were exquisitely specific to the main components of the myocardial sarcomeres. Strong and specific glow after labeling of cardiac myosin strictly coincided with the A-bands (Figure 2B). The labeling was more prominent near the M-line, co-localized with creatine kinase, due to reduced steric hindrance. That was also the case at the A-bands’ edges (Figure 2C). Strong fluorescence labeling of α-actinin sharply co-localized with the Z-line (Figure 2D). That labeling was flanked by highlighted elastic domains of titin in close proximity and on both sides of the Z-line (Figure 2E). Labeling of cardiac actin co-localized with the I-bands, which were intercalated between the A-bands and Z-lines. All the samples were run in triplicates. The images were representative for all the patients’ samples studied.

Strong and specific immunolabeling of the sarcomeric proteins confirmed excellent preservation of antigenicity and molecular architecture, which were essential for anchoring the htAbs upon them.

### Human autologous pluripotent induced stem cells

**Figure 3:**
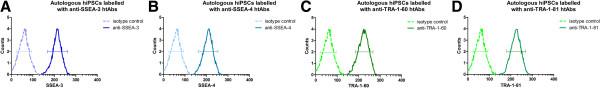
**Flow cytometry of autologous human induced pluripotent stem cells.** Autologous human induced pluripotent stem cells (ahiPSCs) were labeled with the antibodies highlighting the hallmark biomarkers: **A**: anti-SSEA-3; **B**: anti-SSEA-4; **C**: anti-TRA-1-60; **D**: anti-TRA-1-81. The variability in the spectra between the patients was addressed by calculating the full width at half maximum (FWHM) and superimposing over the spectrum.

**Figure 4:**
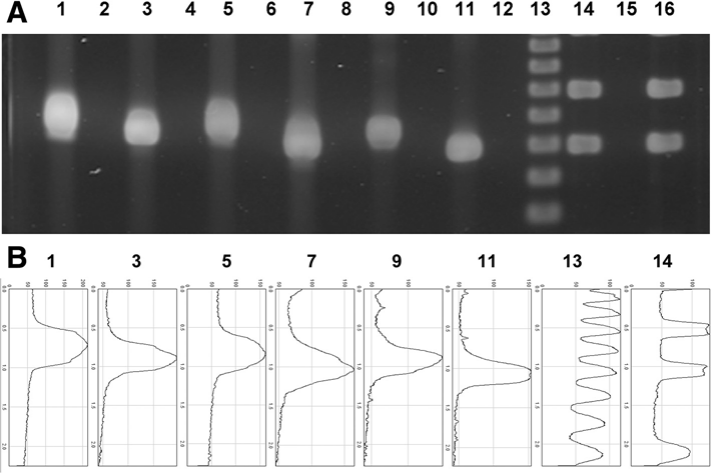
**Electrophoresis of reverse transcribed and amplified ahiPSCs pluripotency genes’ transcripts.** RNA was extracted from the ahiPSCs and PBMCs, reverse transcribed to cDNA, and amplified. **A.** Amplicons were electrophoresed and imaged. **B.** The lanes were quantified. Amplicons on the lanes: 1–4, 3–8, 9–12 for the patients C001-C003 respectively; 13–14, 16 standards; 15 no primers. The amplicons of ahiPSCs on the lanes: 1,3,5,7,9,11 and of PBMCs on the lanes: 2,4,6,8,10,12; *OCT4*: lanes 1–2, 5–6, 9–10, *NANOG*: lanes 3–4, 7–8, 11–12.

**Figure 5:**
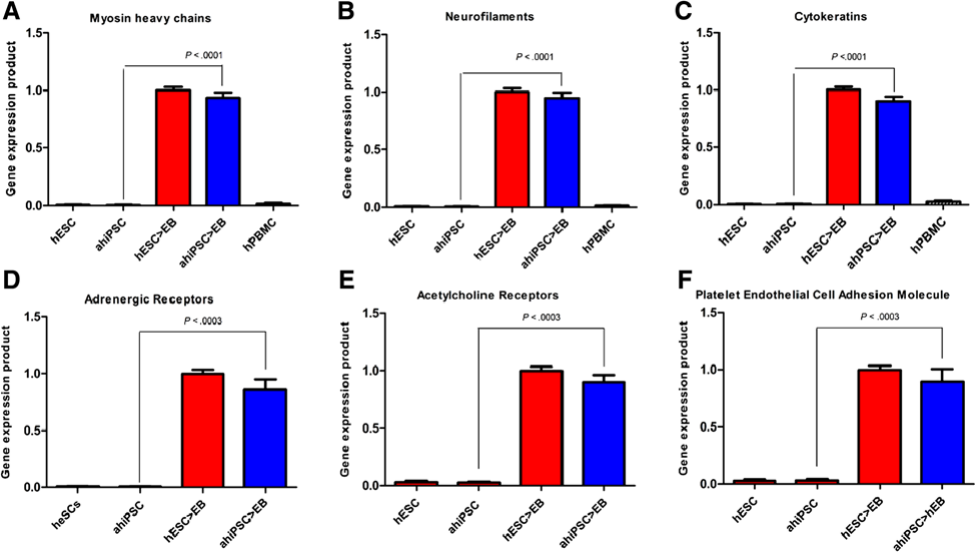
**Differentiation to embryoid bodies from ahiPSCs.** The ahiPSCs’ demonstrated by their ability to grow, while aggregating, into embryoid bodies (EB) and to differentiate into three germ layers with the human embryonic stem cells (hESCs, H1, H9) and the PBMCs included as the controls. It was determined by labeling with superparamagnetic antibodies and measuring relaxivity by NMRS and validated by EDXS, while normalized against total amount of protein. Biomarkers: **A**: myosin heavy chains (MHC); **B**: neurofilamentous proteins (NF); **C**: cytokeratins (CK); **D**: Adrenergic β1 receptor (AdrR); **E**: Acetylcholine receptor (AChR); **F**: platelet endothelial cell adhesion molecules (PECAM).

Peripheral blood mononuclear cells (PBMCs) were transfected with the plasmids DNA of *OCT4, SOX2, NANOG, LIN28, KLF4, MYC* genes. This resulted in efficient reprogramming towards human, autologous, pluripotent, induced stem cells (autologous hiPSCs) as documented (Figures 3, 4 and 5). Outcomes of reprogramming were determined by labeling with the htAbs targeting cell surface displayed biomarkers, which were considered to be the hallmarks of pluripotency: SSEA-4, SSEA-3, TRA-1-60, TRA-1-81. The labeled autologous, human, induced pluripotent, stem cells (ahiPSCs) were analyzed by flow cytometry (Figure 3A-D). Their expression profiles were highlighted as unique for pluripotent stem cells, as validated by isotype controls. All samples were run in triplicates and the ones shown are representative for all. After that validation, batches of expressers were isolated by magnetic and fluorescent activated cell sorting.

Pluripotency of the autologous hiPSCs was also determined based quantification of gene transcripts unique for pluripotent cells: *OCT4* and *NANOG* (Figure 4). Gene expression profiles were determined by qRTPCR followed by electrophoresis of amplicons, and digital image acquisition. Quantification of pixel densities in all lanes facilitated quantification of gene expression, verification of amplification specificity, and exclusion of possible mispriming. The assays were performed as pairs: original PBMCs (Figure 4 2,4,6,8,10,12) before reprogramming and corresponding autologous hiPSCs for every patient (Figure 4 1,3,5,7,9,11). All samples were run in triplicates. As shown, genes *OCT4* and *NANOG* were not expressed in PBMCs, but were strongly expressed in autologous hiPSCs.

Pluripotency of autologous hiPSCs was also validated by their ability to form embryoid bodies (EBs), which differentiated into the three main germ layers with the increasing levels of lineage specific genes, while the hESCs and the PBMCs served as the controls (Figure 5). The EBs were labeled with the synthetic antibodies targeting myosin heavy chains (Figure 5A), neurofilamentous proteins (Figure 5B), cytokeratins (Figure 5C), adrenergic β1 receptor (Figure 5D), acetylcholine receptor (Figure 5E), and platelet endothelial cell adhesion molecules (Figure 5F). Each of the antibodies was modified with superparamagnetic, elemental tags. This feature makes the quantification of the labeling non-destructive and possible on living cells. This also opens the possibilities for monitoring of the processes of differentiation *in vivo*. Furthermore, the measurements were confirmed with energy dispersive x-ray spectroscopy (EDXS) *in situ*. Spectral separation facilitated simultaneous measurements of all three biomarkers in each of the EBs. These measurements finally confirmed solid pluripotency of the generated autologous hiPSCs. Moreover, revealing cell surface display of the receptors transmitting the functional stimuli and the adhesion molecules responsible for interactions with other cells affirmed that the generated cells carry a potential for integrating with the functioning myocardium of the host (study in progress).

**Figure 6:**
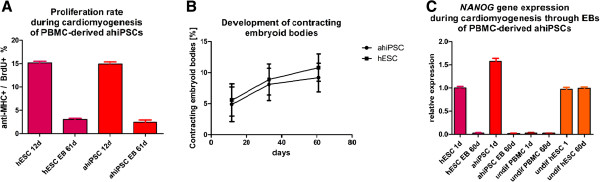
**Proliferation and differentiation rates. A**. Proliferation rates of the ahiPSCs, in the embryoid bodies differentiating towards cardiomyocytes, for 12 days and 61 days, were determined by ratios of bromodeoxyuridine (BrdU) to cardiac myosin heavy chains (MHC) normalized against all cells. **B**. Progressing differentiation was determined by counting the increasing number of embryoid bodies initiated from the hESCs and ahiPSCs. **C**. Progressing differentiation associated with suppression of *NANOG* expression - the key pluripotency gene in the EBs initiated from the hESCs and the ahiPSCs after 1 day (hESC 1d and ahiPSC 1d) versus 60 days (hESC 60d and ahiPSC 60d), while compared to the undifferentiated hESCs and ahiPSCs (undif hESC and undif ahiPSC) as well as the PBMCs (undif PBMC 1d and undif 60d). The statistical significance was accepted for *P* < .05.

Proliferation and differentiation of the cells in the embryoid bodies were studied by measuring incorporation of bromodeoxyuridine (BrdU) (indicator of proliferation) and labeling of cardiac myosin heavy chains (MHC) (Figure 6A). The data indicating progressing differentiation were further supported by the decisive increase in the number of EBs (Figure 6B). The progressing differentiation was associated with suppression of the pluripotency genes – in particular *NANOG* (Figure 6C).

### Recruitment and retention of pluripotent stem cells to infarcted myocardial sarcomeres

**Figure 7:**
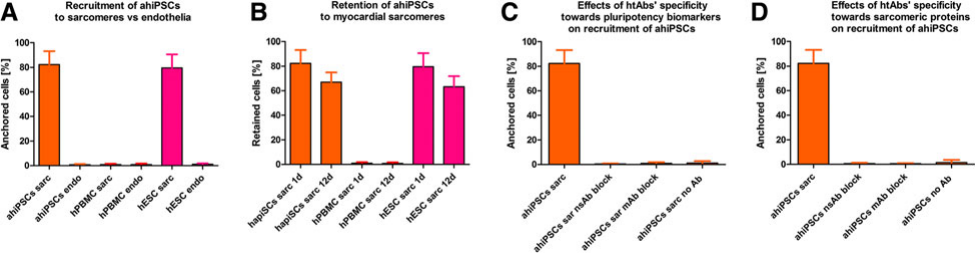
**Recruitment and retention of pluripotent stem cells to infarcted myocardium** ***in vitro.*** The autologous human induced pluripotent stem cells (ahiPSCs) were anchored to infarcted myocardia with the aid of heterospecific, tetravalent antibodies (htAbs). The data were validated with EDXS. **(A)** Efficacy of the htAbs to anchor ahiPSCs to cardiac muscle sarcomeres (ahiPSCs sarc) or endothelium (ahiPSCs endo). The htAbs were constructed from anti-SSEA-4, anti-TRA-1-60, anti- myosin, anti-α-actinin. For comparison, the efficacy of the htAbs to anchor the human cultured embryonic stem cells H1, H9 to the sarcomeres (hESC sarc) and to endothelium (hESC endo) and the PBMCs to the sarcomeres (hPBMC sarc) and endothelium (hPBMC endo) was determined. The measurements for each patient were conducted in triplicates. The data presented here are representative to all the samples studied. The statistical difference was accepted at *P* < .0003. **(B)** Retention of the anchored ahiPSCs onto the sarcomeres upon the day 1 of administration (ahiPSC sarc 1 d) was compared with that after 12 days (ahiPSC sarc 12 d). For reference, retention of the hESCs and the hPBMCs were quantified at the same time intervals. **(C)** Specificity of the htAbs to anchor the ahiPSCs to sarcomeres (ahiPSCs sarc) was tested by blocking the antigens on the ahiPSCs with non-specific tetravalent antibodies (anti-EGFRvIII, anti-EGFRvIV, anti-CEA, anti-PSMA) (ahiPSC sarc nsAb block) and with monospecific antibodies (anti-SSEA-4, anti-SSEA-3, anti-TRA-1-60, anti-TRA-1-81) (ahiPSCs sarc mAb block), or by omitting antibodies altogether (ahiPSCs no Ab). Blocking these biomarkers almost entirely eliminated anchoring of the ahiPSCs to the sarcomeres. The statistical significance was accepted at *P* < .0003. **(D)** Specificity of the htAbs to anchor the ahiPSCs onto the sarcomeres (ahiPSCs sarc) was measured by blocking antigens on the human cardiac muscle sarcomeres with non-specific tetravalent antibodies (anti-EGFRvIII, anti-EGFRvIV, anti-CEA, anti-PSMA) (ahiPSCs sarc nsAb block), with the monospecific antibodies (anti-myosin, anti-actin, anti-α-actinin, anti-titin) (ahiPSCs mAb block), or by omitting antibodies altogether (ahiPSCs no Ab). Blocking of these sarcomeric molecules almost entirely eliminated anchoring of the ahiPSCs to the sarcomeres. The statistical significance was accepted at *P* < 0.0003.

Efficacy of the htAbs’ to recruit and retain human autologous pluripotent induced stem cells (autologous hiPSCs) to infarcted myocardium was assayed in the *in vitro* model and quantified (Figure 7). In these assays, autologous hiPSCs, hESCs, or PBMCs, were injected into buffers, which were circulating through the chamber containing cardiac tissues.

Recruitment of autologous hiPSCs to infarcted myocardium was quantified by counting numbers of the attached cells (Figure 7A). These numbers were determined by changes in relaxation times caused by presence of superparamagnetic tags and registered by NMRS. Alternatively, these numbers were also calculated by changes in scintillation counts caused by elemental tags and registered by EDXS or XRFS. The cell counts were normalized per unit of cardiac tissue mass. All measurements were performed in triplicates for each patient and were representative for all studied, as documented. Numbers of the recruited cells were much higher in assays, in which administration of the htAbs preceded administration of pluripotent stem cells. Numbers of the anchored autologous hiPSCs were comparable to hESCs, which displayed the same biomarkers of pluripotency and served as positive controls. The htAbs had no effect on adhesion of PBMCs, which did not display the biomarkers of pluripotency and served as negative controls. The htAbs did not affect attachment of the autologous hiPSCs to endothelial monolayers.

Retention of the anchored cells was quantified by counting numbers of the cells, which remained detected onto the sarcomeres over different periods of time (Figure 7B). Quantifications were conducted as described above. All pluripotent stem cells, autologous hiPSCs and hESCs, anchored to the sarcomeres with the htAbs, were efficiently retained with minimal losses. As there was practically no recruitment of PBMCs, so was no retention.

Specificity of the htAbs, in recruiting the ahiPSCs to the sarcomeres, was promoted by the htAbs’ domains anti-SSEA-3, anti-SSEA-4, anti-TRA-1-60, or anti-TRA-1-81 (Figure 7C). Introduction of the monospecific antibodies blocking these biomarkers onto the ahiPSCs and hESCs resulted in significant reduction in the numbers of the anchored cells. They practically abrogated anchorage of the autologous hiPSCs. The non-specific antibodies did not enhance recruitment of these cells to the sarcomeres.

On the other hand, specificity of the htAbs for recruiting the ahiPSCs to the sarcomeres relied onto their domains targeting the sarcomeric proteins: anti-myosin, anti-actin, anti-α-actinin, and anti-titin (Figure 7D). Monospecific antibodies and free circulating sarcomeric proteins were used to block antigens on sarcomeres and binding sites of the htAbs (respectively). Blocking the domains on the sarcomeres practically prevented anchoring the htAbs onto them and eliminated their bridging functions for the ahiPSCs.

### Cardiomyogenesis of autologous human induced pluripotent stem cells

**Figure 8:**
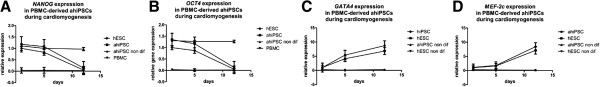
**Cardiomyogenesis of pluripotent induced stem cells.** Differentiation towards myocardial lineage was quantified by measuring levels of gene expression by qRTPCR in human autologous pluripotent induced stem cells (ahiPSCs) (patients C001-C006), human embryonic stem cells (hESCs) (lines H1, H9) – positive control), peripheral blood mononuclear cells (PBMCs) (patients C001-C006) - negative control. In the three time points on the days 1, 5, and 12, the levels of transcripts for: pluripotency genes *NANOG* **(A)** and OCT4 **(B)** decreased, while the levels of transcripts for early myogenesis genes *GATA4* **(C)** and *MEF-2c* **(D)** increased. The statistical significance was accepted for *P* < .05.

Cardiomyogenesis of the stem cells, which were retained onto the myocardial sarcomeres, was initiated by BMP, NAM, and Wnt3. Outcomes of this directed differentiation were determined based upon quantification of gene transcripts (Figure 8). At the selected time intervals, the cells were harvested for mRNA extraction. That followed by quantitative reverse transcription and polymerase chain reaction (qRTPCR). All the patients' samples (C001-C006), positive controls (hESCs: H1, H9), and negative control (PBMCs: C001-C006) were run in triplicates and the data were merged. During the time course of differentiation, the gene expression profiles were changing. Already within 5 days from the first administration of those differentiating factors, the changes were apparent.

Quantities of transcripts for genes characteristic for pluripotency: *OCT4* and *NANOG* were decreasing. Simultaneously, quantities of transcripts for genes unique for myogenesis: *GATA4* and *MEF-2c* were increasing. These trends continued throughout entire periods studied. Identical changes occurred in the hESCs exposed to the same factors. These changes did not occur in the PBMCs.

### Functional tests of contractile apparatus and adrenergic receptors in cardiomyocytes developing from the ahiPSCs

**Figure 9:**
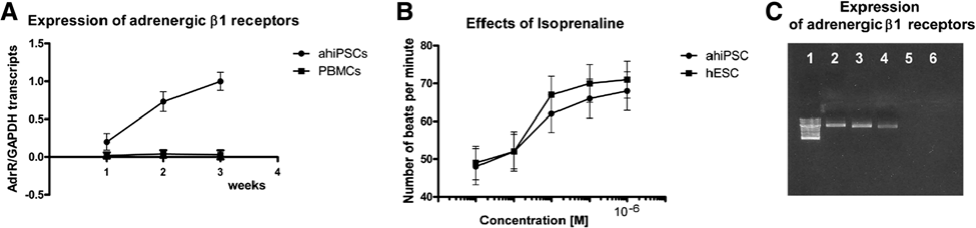
**Functional tests of contractile apparatus and adrenergic receptors in developing cardiomyocytes. A**. Differentiation of cardiomyocytes was determined by qRTPCR of the transcripts for adrenergic β1 (AdrR) receptors normalized by ratios with GAPDH over time. The PBMCs served as the controls. **B**. Functional maturity of the AdrRs was quantified by measuring the beat rate in embryoid bodies after treatment with increasing concentrations of Isoprenaline - adrenergic β1 agonist. **C**. Expression of adrenergic β1 (AdrR) receptors in the differentiating hESCs (lane 4) and ahiPSCs (lane 3) was compared to the cardiac tissue (lane 2) and standard (lane 1), while the undifferentiated ahiPSCs (lane 5) and the PBMCs served as the controls (lane 6).

The autologous human induced pluripotent stem cells (ahiPSCs) were capable of differentiating into the fully functional cardiomyocytes. That was determined by steadily increasing numbers of the adrenergic β1 receptors displayed on the differentiating cells (Figure 9A). The displayed receptors were clearly fully functional as the administration of the Isoprenaline resulted in the significant (almost 50%) increase in the beating rate (Figure 9B). Therefore, in addition to the full functional maturity of the myocytes generated from the ahiPSCs validated by rhythmic contractions – “beating”, the functional maturity of the AdrRs was also validated. Molecular basis for these phenomena were demonstrated by reverse transcription of mRNA into cDNA, nested qRTPCR for the adrenergic β1 receptors, and electrophoresis of amplicons (Figure 9C). It revealed the robust expression of the genes for the adrenergic β1 receptors.

## Discussion

Herein, we report the proof of concept for reprogramming of the patients’ blood mononuclear cells into autologous stem cells, recruiting these stem cells to infarcted myocardium with bioengineered antibodies *in vitro*, and initiating their cardiomyogenesis *in situ*. Below, we discuss several aspects of this novel, but complex therapeutic strategy.

In this study, the patients’ own peripheral blood mononuclear cells were acquired by blood draws. This is surely the least traumatic way for patients to source stem cells for regenerative therapy. In particular, if it is compared to other means of cell acquisition by bone marrow aspiration, cardiac biopsy, or skin excision. Moreover, thinking prospectively about streamlining of this experience into clinical trials, using autologous stem cells reduces risks of potential immune responses to therapeutically delivered cells or side effects due to immuno-suppression. Advantages of reduced risks of immunological response expand also onto other elements of this strategy. All components of cell selection, culture, and antibody engineering were of human origin, which reduced potential risks of immunological response triggered by toll like receptors. In particular, recombinant proteins produced in mice, yeast, or bacteria are well known to trigger such rapid responses. Furthermore, our *in vitro* model of myocardial infarction therapy consisted of all human constituents. This is one of the most important factors in conducting laboratory research in preparation for clinical trials. It is well established that human pluripotent stem cells have different expression profiles, than those from non-humans. Stage-specific embryonic antigen 1 (SSEA-1), which is displayed on mouse embryonic stem cells is absent on early human embryonic stem cells, but is expressed only upon differentiation, is one of many examples. Ignoring these facts results in only less than 0.4% of laboratory research is streamlined to clinical trials [11, 12]. The presented model has its drawbacks in studying long-term changes, which occur during healing processes *in vivo*. These processes may include remodeling of extracellular matrix, fibrinogen-fibrin transitions, mounting immune response, infiltration with immune cells, or continuous influx of cytokines. Nevertheless, refining this fully human *in vitro* model, which we have developed, should improve defining correct starting conditions for myocardial infarction stem cell therapy.

Initial successes in inducing pluripotency of human fibroblasts were attained through delivery of coding sequences for four transcription factors by retroviral vectors. Unfortunately, both, reprogramming factors and retroviral vectors, are potentially cancerogenic. Improvements of those original protocols involved switching to non-viral vectors, reducing numbers of transcription factors, inclusion of small molecules. In particular, some small molecules may reduce thresholds for activation of genes inducing pluripotency. These include valproic acid (VPA) inhibiting histone deacetylase (HDAC), 5-azacytidine (AZA) inhibiting DNA methyltransferase, or E-616452 inhibiting Tgf-β signaling. Although, these small molecules may work to specifically bind to and disable targeted biomolecules, the natural transcription factors not only activate pluripotency cascades, but also repress differentiation signaling. Accordingly, genomic profiling revealed differences between pluripotent cells reprogrammed from adult cells by various protocols, which often explained low efficacies and inconsistencies in abilities of these cells to differentiate. These data prompted us to design pluripotency inducing protocols including six transgenes, which were enhanced by small molecules. We also designed the vectors to host chelating domains saturated with atoms of exogenous metals. This approach facilitated not only efficient induction of pluripotency, but also easy monitoring of the transfected stem cells’ kinetics by XRFS or NMRS [10].

The most serious concern for potential streamlining of the pluripotent stem cells into clinics is the fact that these cells carry inherent risks of giving raise to tumors [41–45]. In fact, one of the cells' pluripotency tests is their ability to form teratomas in nude mice. Clinical cases of tumors grown in hearts as outcomes of stray stem cell therapy were reported [42]. The main strategies have been developed aimed at reducing the risks of iatrogenic tumorigenesis by human induced pluripotent stem cells, as recently reviewed [41, 44, 50]. First, antibodies against the biomarkers displayed on surfaces of undifferentiated cells facilitate sorting them out from populations of differentiating cells. Some of these antibodies demonstrated toxic effects towards undifferentiated stem cells. These effects can be further enhanced by linking toxins to those antibodies on an identical manner as in cancer immunotherapy. Second, differentiated cells after being engineered with reporter genes facilitate sorting them in. Only the precursors of the desired lineages are included into the further steps. The third approach relies upon delivery of the cell suicide inducing genes on a manner identical to cancer suicide gene therapy [46–50].

Retention of pluripotent stem cells to injured zones was defined as the most critical problem for progressing stem cell therapy [5]. Herein, we addressed this problem by bioengineering heterospecific antibodies serving as bridges between stem cells and regenerated myocardium. In the strategy, stem cell recruitment and retention are contingent upon specificity of bioengineered antibodies, as well as strong display of biomarkers by pluripotent stem cells and preservation of antigenicity by sarcomeric molecules. High specificity of the bioengineered antibodies has been attained by gene shuffling and *in vitro* evolution as demonstrated by immunoblotting, multiphoton imaging, NMRS, EDXS, and flow cytometry. Strong and sustained display of biomarkers on stem cells is contingent upon their viability. Therefore, we committed rounds of depletion of apoptotic and dying cells in addition to rounds of positive selection of cells displaying biomarkers of pluripotency [11, 12]. Equally important was securing solid matrix for heterospecific antibodies to attach to. Preservation of architecture and antigenicity of sarcomeres was supported by using the University of Wisconsin Solution. It could be further enhanced by introducing heterospecific antibodies cross-linking sarcomeric proteins, thus to enforce retention of scaffolds. Nevertheless, we are aware of potential risks of stray heterospecific antibodies. They have to be cleared from circulation, so that they cannot cluster multiple cells and create microemboli.

The ultimate goal of this endeavor was to generate new cardiomyocytes *in situ*. Therefore, we designed this project, so that the factors directing differentiation would simulate molecular and temporal patterns, which occurr during human embryogenesis. They would be compatible with human signaling pathways and suitable for future application *in vivo*. That was our preference, rather than relying upon small synthetic molecules like KY02111. Bone morphogenetic proteins 2/4 (BMP) belong to transforming growth factor-β super family, which upon binding to their receptors, trigger SMAD signaling pathways. This leads to formation of mesoderm and progressing through differentiation with expression of *GATA4, Mcef2c, and Serf.* Wnt3 binds to frizzled receptors, while starting signaling pathway, which is modulated by β-catenin. That leads also to mesoderm formation, but later to expression of *Nkx2-5, Isl1, and Baf60c*. These specific factors are further enhanced by inclusion of NAM. In this study, we are primarily focused on initiating cardiomyogenesis. Therefore, the media contained not only supplements stimulating cardiomyogenesis, but also blocking other routes of differentiation by antibodies to VEGFR and PDGFR, which otherwise could trigger differentiation towards endothelia and smooth muscle (respectively). Ahead of us, is to define temporal and molecular patterns, which are not only initiating, but also promoting final stages of differentiation including formation of gap junctions and integration with healthy myocardium.

From the cardiothoracic surgeons’ point of view, this approach of stem cell therapy of infarcted myocardium should be most effective, if applied at early periods of time after infarction. This would be best at the time of or immediately after revascularization [3, 4]. At those times, the htAbs would have best access to the exposed sarcomeric proteins. With passing times, healing scars create barriers for antibodies and cells. Moreover, molecular components of scarred tissue are different, than those at early stages of infarction. In particular, production of collagen creates different matrix for stem cells to interact with. Influx of cytokines also affects stem cells. Therefore, specificity of bridge antibodies and repertoire of cardiomyogenesis stimulating molecules may have to be adjusted in relation to changing with time healing processes *in situ*. This strategy will have to be refined even further to take into account patients’ medical histories and genomic profiles.

From hospital pharmacists’ point of view, developed heterospecific antibody biotechnology is very robust. The assembled htAbs have solid stability with a long shelf due to high affinity constants between components (biotin-avidin bonds) [15, 16]. As such, this biotechnology can be easily adapted to GMP standards within cardiothoracic surgery departments. Moreover, this biotechnology is very flexible. Therefore, if new biomarkers of autologous stem cells are discovered, then specific antibodies can be selected from the human antibody library and easily incorporated into this antibody platform; thus into this therapeutic strategy.

For the purpose of our studies on regeneration, we have developed means to quantify processes not only *in vitro* in research labs, but also *in vivo* in clinical settings. The keystones of this approach are the superparamagnetic antibodies, which we designed, synthesized, and thoroughly characterized previously [14–18]. The main advantage of these antibodies is that they facilitate precise quantification of these processes not only in vitro by NMRS, but also they are directly applicable in vivo with MRI. Moreover, they are safe to use in a teaching institutions setting. The acquired data are validated with EDXS and XRFS. Finally, the data are instantly available and can be used for adjusting the protocols. An alternative approach would involve using antibodies charged with radionuclides. After screening by scintillation counting, it could be extrapolated to PET, SPECT, or Gamma. However, this option requires special infrastructure and special training of the investigators, as well as greater potential risks and side effects for patients and staff and students. An alternative approach would involve fluorescent derivative of antibodies. However, this approach is so far only limited to studies on cells or sections with a very limited possibility for extrapolation into clinics due to hard to deconvolve scattering of light by the tissue. Therefore, in this study we relied primarily on the synthetic antibodies tagged with atoms of the exogenous elements rendering them super-paramagentic.

In this study, we demonstrated that we can significantly enhance recruitment and retention of the autologous human induced pluripotent stem cells (ahiPSCs) to the infarcted myocardium with the aid of the heterospecific tetravalent antibodies (htAbs). We decided to choose this route with potential streamlining into the clinics. Moreover, we did so *in lieu* of reports of rejection of heterologous transplants. Nevertheless, allogeneic sources may be considered.

We have completed this work on the ahiPSCs. While we improved the recruitment and retention of the stem cells, the first and foremost problem in using any pluripotent stem cells in clinics is the risk of their inherent tumorigenicity. While initial screening prior to implantation may facilitate elimination of the tumorigenic clones, the strategies allowing us to monitor processes of differentiation *in vivo* after implantation of therapeutic cells, as well as to eliminate instantly any cells heading towards tumorigenic transformation are really necessary as recently reviewed [46–50]. In addition, two strategies may further enhance safety of the stem cell therapy, while relying on the enhanced stem cell recruitment biotechnology presented herein: direct trans-differentiation and induction of progenitors. The direct trans-differentiation relies upon lineage reprogramming with the defined factors [10, 28–38]. Therefore, the pluripotency stage is entirely eliminated. In this strategy, the htAbs’ domains anchoring onto the infarcted myocardium sarcomeres remain the same, but those domains anchoring therapeutic cells are modified to tag the cell surface displayed biomarkers. This strategy facilitated significantly enhanced recruitment and retention of the clones of bone marrow cells expressing CD34, CD117, and CD133 to the sarcomeres of the infarcted myocardia [10]. Thereafter, the differentiation is propelled by the homing environment and additional factors inducing the directed lineage reprogramming. Yet another strategy relies upon reprogramming adult cells into human induced pluripotent stem cells followed by their differentiation towards desired lineage progenitors [22–24]. For the purpose of this strategy, the htAbs’ domains anchoring onto the infarcted myocardium sarcomeres remain the same, but those domains anchoring the progenitors are modified to the specific cell surface display profiles. We currently pursue both routes.

## Conclusion

The proof of concept has been attained *in vitro,* for reprogramming the patients’ blood mononuclear cells into human, autologous, pluripotent stem cells, recruiting these stem cells to infarcted myocardium, and initiating their cardiomyogenesis. This novel strategy is ready to support the ongoing clinical trials in cardiothoracic surgery aimed at regeneration of infarcted myocardium.

## Abbreviations

PBMCs: Peripheral blood mononuclear cells
autologous hiPSCs: Autologous human induced pluripotent stem cells
ahiPSC: Autologous human fragment antibody
scFv: Single chain variable fragment antibody
dcAb: Dual chain variable fragment antibody
Oct4 aka POU5F1: Octamer-binding transcription factor 4A aka POU-type homeodomain – containing DNA binding protein
Sox2: SRY (sex determining region Y)-box 2 factor
Nanog: Homeobox transcription factor
Lin28: Cytoplasmic microRNA binding protein
Klf 4: Kruppel-like factor 4
SV40: Simian vacuolating virus 40
SSEA-4: Stage specific embryonal antigen 4
SSEA-3: Stage specific embryonal antigen 4
TRA-1-60: Tumor related antigen recognized by the monoclonal antibody 1-60
TRA-1-81: Tumor related antigen recognized by the monoclonal antibody 1-81
FCM: Flow cytometry
IB: Immunoblotting
MACS: Magnetic activated cell sorting
FACS: Fluorescent activated cell sorting
NMRS: Nuclear magnetic resonance spectroscopy
TRXFS: Total reflection x-ray fluorescence spectroscopy
MPFS: Multiphoton fluorescence spectroscopy
EELS: Electron energy loss spectroscopy
EDXS: Energy dispersive x-ray spectroscopy.
